# Exogenous Melatonin Improves Fruit Quality Features, Health Promoting Antioxidant Compounds and Yield Traits in Tomato Fruits under Acid Rain Stress

**DOI:** 10.3390/molecules23081868

**Published:** 2018-07-26

**Authors:** Biswojit Debnath, Mubasher Hussain, Min Li, Xiaocao Lu, Yueting Sun, Dongliang Qiu

**Affiliations:** 1 College of Horticulture, Fujian Agriculture and Forestry University, Fuzhou 350002, China; biswo26765@yahoo.com (B.D.); mubasherhussain05uaf@yahoo.com (M.H.); liminzyl@sina.com (M.L.); xc531599541@126.com (X.L.); yuetingsun@126.com (Y.S.); 2 Department of Horticulture, Sylhet Agricultural University, Sylhet 3100, Bangladesh

**Keywords:** simulated acid rain, melatonin, bioactive compounds, antioxidants, *Solanum lycopersicum* L.

## Abstract

Acid rain is a serious worldwide environmental problem which reduces the growth and yield of crops. Melatonin, as a pleiotropic molecule has been known to improve stress tolerance by limiting the oxidative damage of plants exposed to adverse environments. However, the role of exogenous melatonin particularly on the yield and antioxidant compounds in tomato fruits under abiotic stress condition remains inexpressible. This observation aims to identify the influence of melatonin treatment under simulated acid rain (SAR) condition on fruit qualities, phenolics, flavonoids, and carotenoids concentration in fruits, and yield of tomatoes. Our study results showed that the fruits of SAR-stressed plants had higher quality traits and antioxidant bioactive compounds by increasing antioxidant activities against SAR-induced oxidative stress compared with fruits of control plants. Nonetheless, these improvements to antioxidant activities in fruits under SAR-condition remained unable to prevent the reduction of the yield. However, SAR-stressed plants treated by melatonin exhibited upgradation on the fruit quality traits, antioxidant compounds and yield attributes through accelerating oxidant-scavenging antioxidant actions in fruits compared with fruits of SAR-stressed plants. Meanwhile, our results suggest that exogenous melatonin plays an important role in improvement of bioactive compounds and yield traits in tomato fruits through regulating antioxidant system.

## 1. Introduction

Fruits and vegetables provide us with energy, vitamins, minerals, and fibers, which are considered to be important elements of a healthy and balanced diet. Therefore, they are necessary as part of a part of main meal or as beverage additives. Moreover, they contain some important minor components which are called phytochemicals or secondary plant metabolites (phenolics, flavonoids, and carotenoids), which not only give different pigments to fruits and vegetables but are potentially beneficial for our health [1,2]. Different phytonutrients in a healthy diet having the potential to lower the cardiovascular disease and certain cancers, moreover they have antioxidant, anti-inflammatory and anti–tumor properties [3,4,5]. However, these important phytonutrients are inadequate in the edible parts of most commonly consumed fruits and vegetables worldwide, like the tomato, as compared to blueberry, rubus berries, cheery, eggplant peel, grape, black carrot and red cabbage [6,7].

Tomato fruits are universally documented as a health promoting diet and considered as a good source of balanced nutrition [8]. The tomato is cultivated widely for fresh consumption or processing and nowadays, it considered as a core vegetable crop in the world. In 2014, according to the Food and Agriculture Organization (Rome, Italy) estimation, tomato cultivated was about 5.02 million hectares of land in worldwide, and production was 170.75 million tons, among that China produced 30.88% of this total but area was about 19.92% [9]. Due to high yield and profitability, in recent years, the tomato has become one of the major vegetables grown in China [9]. On the other hand, tomato is one of the common food items in Europe and Mediterranean diet. Improvement of nutritional value, as well as yield of tomato fruits is decisive but the different factors such as agronomical practices, environmental condition strongly affects the growth of tomato fruits [10].

Environmental stress has potentially harmful consequences on agricultural production and fruit quality. Undiscerning and continuous uses of energy may cause degradation of natural resources widely and also influence our life care system. Acid rain (AR) is considered to be one of the major environmental issues in recent decades and has been a critical concern in Asia, Europe and North America [11,12]. Sulfur dioxide (SO_2_) and nitrogen oxides (NO_x_) are the major apparatuses of air pollution which emitted from fossil fuels and industrial sources, and those are converted to sulfuric (H_2_SO_4_) and nitric acid (HNO_3_) in precipitation, which ultimately persuaded AR [13]. In Southern China, the regular acid rain causes acidified in the huge area of soils and have an excessive negative impact on the key ecosystem due to rapid economic development [14].

It has been reported that the changes in physiological and biochemical processes of plants such as nutrient loss from leaves, distorted water balance, variation of several antioxidant enzymes occurred because of acid rain deposition at several levels [15,16]. Moreover, the increased production of reactive oxygen species (ROS) molecules inhibited the physiological and developmental aspects in SAR stressed plants as categorized by the increasing membrane damage (lipid peroxidation), degrading the soluble proteins, carbohydrates, and pigment components such as chlorophylls and/or carotenoids contents [17,18]. In plants, the different antioxidant activities have been found higher in simulated acid rain stress condition to prevent accumulation of toxic ROS and membrane damage compared with control plants [19,20]. Usually, the effects of acid rain on biochemical processes may be detected much earlier than visible injuries and noticeable changes in growth and yield since the later become apparent only after exposure to relatively long periods. However, the yield components of tomato fruits have been testified to be negatively affected by the increasing acidity of simulated acid rain [21]. In contrast, the positive responses in tomato fruits quality such as soluble solid, sugar, protein, titratable acid, and different antioxidant enzymes and non-enzymes have been observed under different environmental stress conditions [22,23,24]. Nowadays, improvement of tomato fruit quality as well as yield under abiotic stress condition is a challenge for tomato growers. Moreover, tomato is a typical plant for Solanaceae’s crop and advances in its fruit quality, nutritional attributes and agronomical practices under environmental stress condition may helpful to emerging new approaches for other crops [25].

Melatonin (*N*-acetyl-5-methoxytryptamine) is an indolic compound resulting from the vital amino acid tryptophan structurally related with indole-3-acetic acid (IAA), broadly present in alive organisms covering from bacteria to mammals [26]. It was hypothesized that melatonin act as the first line defense to protect plants against biotic and abiotic stress, and the mechanisms of melatonin-mediated stress tolerance are involved in arranging biosynthesis of antioxidants, activating related enzyme activities and scavenging ROS directly when plants are exposed to harsh environments such as extreme temperature, cold, drought, salt and oxidative stress [26,27,28]. Moreover, our previous studies exposed that exogenous melatonin improved the photosynthesis systems and ROS-scavenging antioxidant activities which lead to enhanced the SAR-stress tolerance in tomato plants [29]. Currently, it has been demonstrated that the presence of melatonin in different parts of plants such as the roots, stems, leaves, flowers, fruits, and seeds [27]. In addition, it was reported that the distribution and accumulation of melatonin during fruit development stage was observed in Micro-Tom, a model cultivar of tomato (*Solanum lycopersicum* L.) [30], and the considerable amounts of melatonin content in tomato fruit were enhanced from mature green stage to red stage especially in pericarp [31,32]. However, the role of exogenous melatonin in the improvement of nutritional values, antioxidant compounds and yield of tomato fruits under environmental stress condition is not well understood.

To well understand the impact of exogenous melatonin in the enhancement of tomato fruit production as well as nutritional values under simulated acid rain condition, we sought to observe in different nutritional compounds, antioxidant enzymes and non-enzymes, and membrane damage in tomato fruits. We hypothesize that exogenous melatonin might have a potential impact on fruits quality, antioxidant compounds and yield in tomatoes under simulated acid rain condition by inducing different antioxidant levels and synthesis of bioactive compounds in fruits. To affirm this hypothesis, we fixed an experiment on *Solanum lycopersicum* L. cv. Micro-Tom as an experimental typical plant. The current observation elicited the conceivable influences of exogenous melatonin in tomato fruits under SAR-stressed condition, which can offer a probable recommendation to maintaining food security predominantly in peripheral area where environmental stress is a restrictive factor.

## 2. Results and Discussion

### 2.1. The Impact of Exogenous Melatonin on Fruit Qualities under SAR-Stress Condition

**Figure 1:**
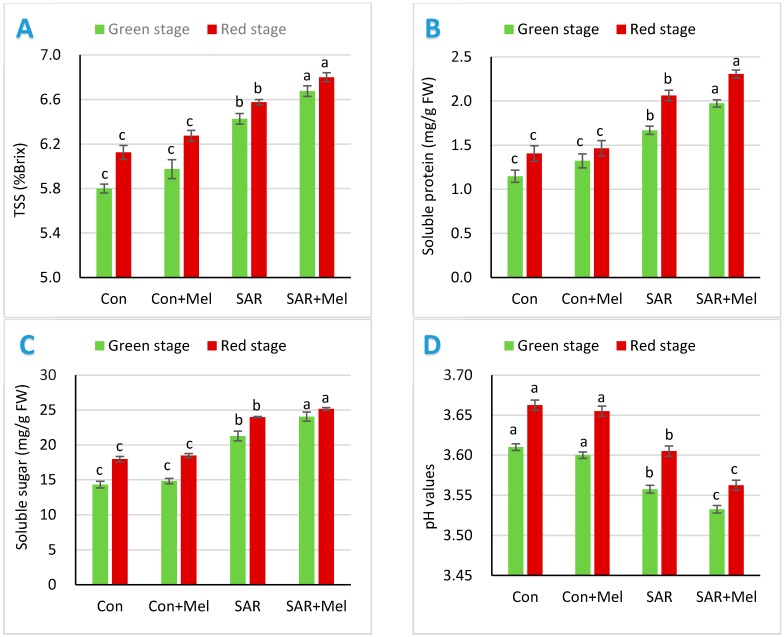
Effect of exogenous melatonin on (**A**) TSS; (**B**) soluble protein; (**C**) soluble sugar; (**D**) pH values in tomato fruits under SAR condition. Values indicate the averages of four replicates and standard error implied by vertical bar. Different letters within the same color of a graph indicates significantly different at *p* ≤ 0.05 (Tukey’s test). Here, FW, Con, Mel, and SAR indicates fresh weight of fruit, control, melatonin, and simulated acid rain, respectively.

To observe the impact of exogenous melatonin treatment under SAR condition on tomato fruit qualities, we estimated the total soluble solid, soluble sugar, soluble protein, pH values in tomato fruits. The amount of TSS, soluble sugar, soluble protein content in fruits determines the sensory characteristics mainly taste and sweetness. The pH value considered to be the consistent parameter of fruit quality and the critical standards in processing industry as they influenced in the shelf life of fruits. As shown in Figure 1, the values of TSS, soluble sugar, soluble protein were increased significantly by 10.86%, 48.74%, 45.22%, respectively in SAR-stressed plants at mature green stage where as these values were increased by 7.34%, 35.59%, 46.1%, respectively in red stage of tomato fruits compared with those in control plants. However, pH values of tomatoes were decreased significantly by 1.39% and 1.37% at mature green stages and red stage, respectively compared with fruits of control plants (Figure 1). The several previous studies observed that abiotic stress boosted the sensory attributes by increasing the TSS, soluble sugar, soluble protein content and decreasing the pH values in tomato fruits [10,22]. Also, supplementation of exogenous melatonin to SAR-stressed plants resulted obvious increased the fruit quality attributes compared to SAR-stressed plants. The TSS, soluble sugar and soluble protein contents in mature green fruits of SAR-stressed plants treated with melatonin increased by 3.89%, 13.12% and 17.96%, respectively, and in red fruits of these values were increased by 3.34%, 4.88% and 12.14%, respectively compared with fruits of SAR-stressed plants (Figure 1). However, the pH values were further decreased significantly in fruits of SAR and melatonin both treated plants compared with the plant exposed to SAR (Figure 1). Whereas, the melatonin supplementation in control plants showed insignificant differences on these fruits quality attributes compared with fruits of control plants (Figure 1). Previous studies reported that melatonin improved fruits quality during fruit development and ripening stages by reduction of cell wall degradation and intercellular adhesion [31]. The findings of our study showed that melatonin improves the fruit quality attributes under SAR stress condition.

### 2.2. The Impact of Exogenous Melatonin on Antioxidant Enzymes in Fruits under SAR-Stress Condition

**Figure 2:**
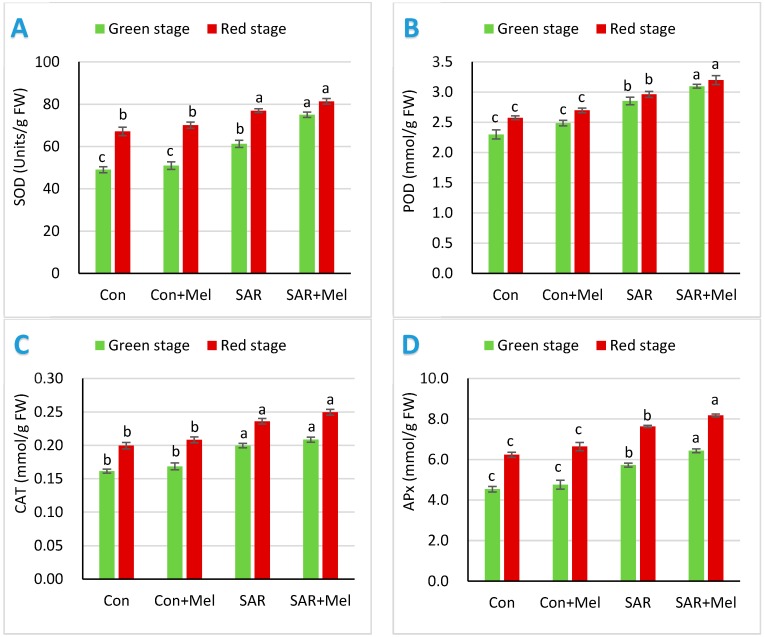
Effect of exogenous melatonin on enzymatic antioxidant activities in tomato fruits under SAR condition. (**A**) Superoxide dismutase; (**B**) guaiacol peroxidase; (**C**) catalase activity; (**D**) ascorbate peroxide. Values indicate the averages of four replicates and standard error implied by vertical bar. Different letters within the same color of a graph indicates significantly different at *p* ≤ 0.05 (Tukey’s test). Here, FW, Con, Mel, and SAR indicates fresh weight of fruit, control, melatonin, and simulated acid rain, respectively.

To identify the SAR-induced changes in the enzymatic antioxidative system of tomato fruits and the role of exogenous melatonin in these respects, we evaluated SOD, POD, CAT and APx activities at mature green and red stage of tomato fruits. The current experiment presented that the activities of SOD, POD, CAT, and APx in fruits of tomato plant at mature green and red stage both were significantly increased by SAR condition compared with fruits of control plants (Figure 2). SAR stressed may cause the impairment of tomato fruits which can be lessened by enhancing the activities of antioxidants compounds to reduced oxidative damage. The results of our study showed that like other parts of plants, tomato fruits also increased the accumulation of reactive oxygen species scavenging enzymatic antioxidant compounds to overcome oxidative damage occurred by SAR stressed compared with control condition (Figure 2). The findings of our study agreed with previous study that antioxidant enzymes in fruits of tomato might have a great role to improve protection mechanism by reducing oxidative and membrane damage under different environmental stress condition [23,24]. Interestingly, melatonin supplementation in SAR-stressed plants caused increased evidently the accumulation of SOD, POD, CAT and APx contents in fruits compared with those in fruits of plants exposed to SAR (Figure 2). Meanwhile, our results indicated that melatonin treatment in SAR-stressed plants served as an indicator to enhance defense system by more rising the accumulation of the ROS-scavenging antioxidant enzymes in tomatoes compared with fruits of SAR alone treated plants. These consequences supported with our previous and other researcher’s studies that melatonin may have an important role to protect the plants by reducing oxidative damage against different abiotic stress conditions [26,29].

### 2.3. The Impact of Exogenous Melatonin on Oxidative Damage and Total Antioxidant Potential in Fruits under SAR-Stress Condition

**Figure 3:**
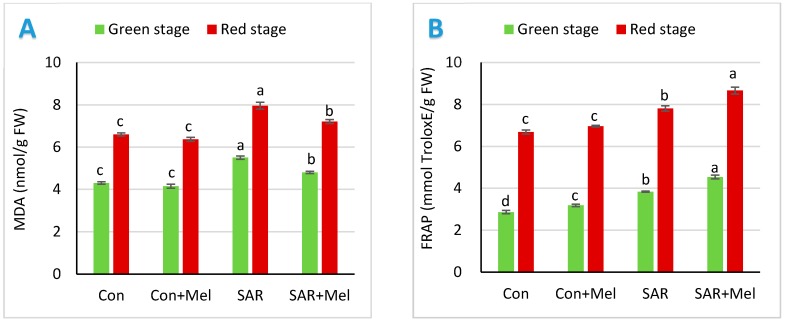
Effect of exogenous melatonin on (**A**) malonaldehyde (MDA) and (**B**) ferric reducing antioxidant power (FRAP) in tomato fruits under SAR condition. Values indicate the averages of four replicates and standard error implied by vertical bar. Different letters within the same color of a graph indicates significantly different at *p* ≤ 0.05 (Tukey’s test). Here, FW, Con, Mel, and SAR indicate fresh weight of fruit, control, melatonin, and simulated acid rain, respectively.

To observe oxidant stress under different treatment conditions, we measured malondialdehyde contents in tomato fruits. MDA was found in plant parts when ROS directly affected normal cellular functioning and irritated oxidative stress through lipid derived radicle’s production. The results in Figure 3A showed that SAR treatment caused increases the concentration of MDA in tomato fruits by 28.14% and 20.61% at mature green and red stage of tomato fruits, respectively compared with the fruits of control plants. It has been shown that the tomato plants exposed to environmental stress caused oxidative damage resulted in higher lipid peroxidase accumulation in fruits [24]. In contrast, application of melatonin in SAR stressed plants showed 12.89% and 9.42% lower MDA contents in mature green and red stages of fruits, respectively compared with fruits of SAR treated plants (Figure 3A). The previous study revealed that melatonin act as a first line defense against oxidative damage in plants and directly scavenges ROS by elevating the ROS-scavenging antioxidant enzymes resulting decreased the concentration of MDA in plants exposed to abiotic stress [26].

To verify the protective impact of melatonin against SAR-induced oxidative damage and total antioxidant activities, we analyze ferric reducing antioxidant power in tomato fruits. The total reducing antioxidant potential characterizes the capacity to inhibit the oxidation process under different stress condition. Compared with the fruits of control plants, SAR exposure increased sharply fruits’ antioxidant power by 34.27% and 16.74% at mature green and red stages, respectively (Figure 3B). The results indicated that fruits activated their antioxidant system against oxidative stress derived by SAR stresses. Similar findings have been reported in another research which revealed that the total reducing antioxidant power increased by activating antioxidants to relieved stress-induced oxidative damages in fruits [33]. Moreover, the melatonin supplementation to SAR-stressed plants causes further increase in the antioxidant power in fruits by 18.23% and 10.88% at mature green and red stages, respectively compared with the fruits of SAR-stressed plants (Figure 3B). Meanwhile, our findings agreed with another study that exogenous melatonin advanced the stress tolerance by inducing their antioxidant potential against oxidative stress [34].

### 2.4. The Impact of Exogenous Melatonin on Phenolics and Flavonoids in Fruits under SAR-Stress Condition

**Figure 4:**
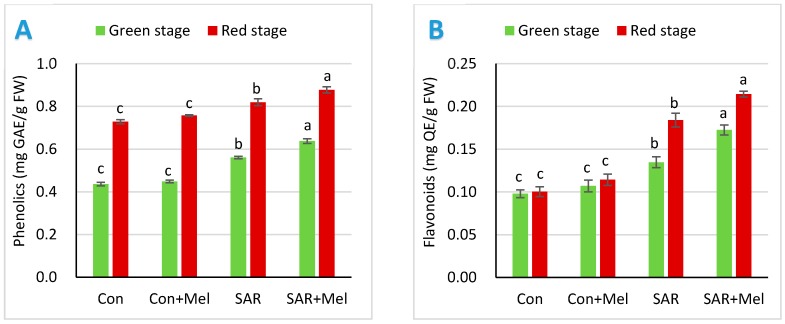
Effect of exogenous melatonin on (**A**) phenolics and (**B**) flavonoids content in tomato fruits under SAR condition. Values indicate the averages of four replicates and standard error implied by vertical bar. Different letters within the same color of a graph indicates significantly different at *p* ≤ 0.05 (Tukey’s test). Here, FW, Con, Mel, and SAR indicates fresh weight of fruit, control, melatonin, and simulated acid rain, respectively.

To explore the impact of melatonin treatment on health promoting antioxidant compounds in tomato fruits against SAR stress, we assessed phenolics and flavonoids contents in fruits. The phenolic and flavonoids acted as a non-enzymatic antioxidant compound under stress condition and considered as important antioxidants in human nutrition [35]. These health promoting bioactive compounds improved the protection capacity of human against thrombosis and tumorigenesis [36]. As shown in Figure 4, the total phenolic and flavonoids content in fruits were increased in fruits of SAR-stressed plants by 27.27% and 30%, respectively at mature green stage, and 12.33% and 80%, respectively at red stage compared with those in the fruits of control plants. These findings agreed with other results that the plant enhances the non-enzymatic antioxidants like phenolic and flavonoids to eliminate oxidative stress when plants exposed to abiotic stress [33,37]. In addition, compared with the fruits of SAR-stressed plants, the phenolic and flavonoids contents were leading to increases of 14.29% and 30.77%, respectively at mature green stage and 7.32% and 16.67%, respectively at red stage of fruits with melatonin supplement in SAR-stressed plants (Figure 4). The consequences of our results supported the perception that exogenous melatonin plays a significant role to enhances the plants defense system in environmental stress condition by uplifting ROS-scavenging antioxidant non-enzymes against oxidative damage [38].

### 2.5. The Impact of Exogenous Melatonin on Carotenoids in Fruits under SAR-Stress Condition

β-carotene and lycopene are the major carotenoids in tomato fruits and act as a potent bioactive antioxidant compounds to constrain tumor cell growth which lead to defend against prostate and other cancers [36]. Carotenoids are one of the prominent pigments for coloration of tomato fruits and considered as an important phytonutrient in a healthy diet. To investigate the impact of exogenous melatonin on pigment concentrations in tomato fruits under SAR-stress condition, we determined β-carotene, and lycopene in red fruits. The concentration of β-carotene and lycopene were increased by 32.74%, and 20.55%, respectively in the fruits of plants exposed to SAR compared with those in the fruits of control plants (Table 1). The finding of our current study agreed with previous study that the accumulation of carotenoids contents in tomato fruits were increased by abiotic stress condition [39]. Moreover, the application of melatonin in SAR-stressed plants caused further significant increases of β-carotene and lycopene contents by 9.31%, and 10.48%, respectively in fruits compared with SAR alone treated plants (Table 1). Meanwhile, our results indicated the exogenous melatonin act as a promoter in biosynthesis of carotenoids in fruits in SAR stress condition. Meanwhile, the findings of our experiments agreed with other’s concept that exogenous melatonin might have an important role in mediating pigment compound accumulation in tomato fruits [31].

### 2.6. The Impact of Exogenous Melatonin on Yield Attributes of Tomatoes under SAR-Stress Condition

**Figure 5:**
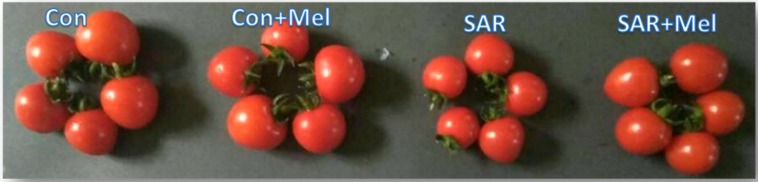
Changes of tomato fruit’s size in SAR and/or exogenous melatonin treatments. Here, Con, Mel, and SAR indicates control, melatonin, and simulated acid rain, respectively.

To evaluate the exogenous melatonin’s impact in SAR-stress condition, yield attributes of tomatoes were estimated. Yield traits reduction indicates the serious symptom of plants those are exposed to any environmental stress. As shown in Table 2 and Figure 5, the values of fruit’s diameter, fruit’s length, number of fruits per plant, and average weight of a fruit were reduced by 23.65%, 23.74%, 52.85%, and 37.20%, respectively but dry matter percentages of fruits increased by 13.76% in plants exposed to SAR compared with those in control plants. Our results agreed with previous observation that the yield components were dramatically reduced by the application of simulated acid rain in plants [19,21]. However, supplementation of exogenous melatonin to SAR-stressed plants exhibited visible improvement of these yield attributes by 15.25%, 16.74%, 68.13%, 34.94%, and 4.09%, respectively compared with SAR-stress plants (Table 2). However, insignificant improvements were observed in between the fruits of control plants and control plants treated with melatonin treatments (Table 2). Meanwhile, the findings of our experiments indicated that exogenous melatonin significantly improved the reduction in yield components of tomatoes when plants exposed to SAR condition. Moreover, this consequence agreed with the concept of other researcher that the spraying of melatonin boosted the stress tolerance of plants which helps in possible mitigation of yield and yield components under abiotic stress condition [26].

## 3. Materials and Methods

### 3.1. Plant Materials and Treatments

The experimental site and weather conditions were described in our previously experiment [29]. *Solanum lycopersicum* L. cv. Micro-Tom tomato seeds were grown in seed pot contained a mixture of vermiculite, peat, and perlite (1:2:1, *v*/*v*) in greenhouse. Uniform seedlings at the three true-leaf conditions were transplanted into a greenhouse pot (24 cm × 14 cm × 15 cm) containing a mixture of vermiculite, cover soil and perlite (1:1:1).

To examine the impact of exogenous melatonin in fruit quality attributes, health promoting key antioxidant compounds and yield of tomatoes against SAR-stress condition, tomato plants at fifth true leaf stage were sprayed by SAR of pH 2.5 and pH 5.6 for alternating days at 4:00 p.m. up to fruit set stage, while SAR of pH 5.6 were used as a control [20]. Simulated acid rain of pH 2.5 and 5.6 were prepared by using sulfuric acid and nitric acid in accordance to our previous experiments [20]. To perceive the influence of exogenous melatonin in fruits of control and stressed plants, 100 µM melatonin was sprinkled in leaves of both control and SAR-stressed plants based on previous research findings [29] for every three days up to fruit set stage. Every treatment consisted of four replicates and a biological replicate comprised of 12 bulked plants.

The fruits of second to fourth tresses from tomato plants harvested at two ripening stags—mature green stage and red stage for different analysis. These mature green and red stages were chosen to represent tomatoes for fresh consumption and processing, respectively [8]. Freshly collected fruits cleaned, sampled and immediately freezing in liquid nitrogen, and at that time kept in refrigerator (−80 °C).

### 3.2. Measurement of Fruit Quality

Total soluble solid (TSS) were quantified by a digital refractometer (DR301-95, Krüss Optronic, Hamburg, Germany) and results expressed as % Brix. The pH values of the fruit samples were quantified using a pH-meter (Orion 828 acidity analyzer, Chelmsford, MA, USA).

The soluble protein content in fruits were estimated based on the method found in [40]. In brief, fruit samples of 0.5 g were homogenized with polyvinylpolypyrrolidone (PVPP) and Tris buffer. The extracts were centrifuged at 14,000× *g* for 20 min at 4 °C. Hence, the supernatants (0.1 mL), and Bradford reagents (3 mL) were added in test tube and keep it for 5 min for mixing. The absorbances were taken against blank at 595 nm in spectrophotometer. The total soluble protein contents of the fruit samples were computed from preparing a calibration curve by bovine serum albumin.

The soluble sugar contents were measured according to anthrone reagent assay where glucose was used for preparing calibration curve [41]. Briefly, fresh fruit samples (0.5 g) were standardized in 70% alcohol (5 mL) and then, water bath for 30 min at 70 °C. Centrifuge the solution at 8000× *g* for 10 min at 20 °C. Take out the supernatant in another test tube and repeat the procedure. Again, take out the supernatant in test tube and made final volume up to 10 mL by 70% alcohol. 0.2 mL supernatant mixed with 5 mL anthrone reagent (anthrone with sulfuric acid) and 0.8 mL distilled water. These mixtures were heated on water bath at 95 °C for 10 min and then ice-cooled. The absorbances were observed from spectrophotometer at 620 nm against blank.

### 3.3. Estimation of Enzymatic Antioxidant Activities, Oxidative Damage and Total Antioxidant Power

Fruit samples (1.0 g) were homogenized in potassium phosphate buffer (50 mM; pH 7.8) and the extracted samples were centrifuged at 12,000× *g* for 20 min at 4 °C. The supernatants were separated and kept in refrigerator at 4 °C for enzymatic assay. These supernatants were used to assess the superoxide dismutase activity (SOD), guaiacol peroxidase (POD), catalase activity (CAT) and ascorbate peroxide activity (APx). The activities of these enzymes were determined by previously described methods [42].

Malondialdehyde contents (MDA) were estimated for the observation of oxidative damage in fruits according to the TBA test [42]. Briefly, 1.5 mL of enzyme extract were homogenized in 2.5 mL of 5% (*w*/*v*) trichloro-acetic acid (TCA) containing 0.5% thiobarbituric acid (TBA) and centrifuged at 4800× *g* for 10 min. 

The total antioxidant potential of the fruit samples were measured by the FRAP (ferric reducing antioxidant power) assay [43] where, the concentrated blue color form due to the TPTZ (2,4,6-tri-pyridyl-triazine)-Fe^3+^ structure change to TPTZ-Fe^2+^. The absorbances of the color solutions were estimated by spectrophotometrically (vs the blank) at 593 nm. The standard curve was prepared by using 1 mM Trolox (6-hydroxy-2,5,7,8-tetramethylchroman-2 carboxylic acid) standard solution. The result was expressed as mmol TroloxE in 1 g fresh weight of fruits.

### 3.4. Quantification of Phenolics and Flavonoids Content

The fresh fruit samples of measuring 1.0 g were homogenized with chilled 80% ethanol (6 mL). After that, the ethanolic extracts were centrifuged at 12,000× *g* for 20 min at 4 °C. These supernatants were used for estimation of the phenolics and flavonoids contents in fruits according to our previous experiments [29].

For total phenolic content analysis, 1.0 mL extract mixed with 0.75 mL Folin-Ciocalteu reagent, 0.25 mL 7.5% Sodium carbonate and 1.0 mL distilled water in a test tube. The mixtures were allowed to incubate for 90 min in water bath at 30 °C. Later, an intense blue color was developed. The absorbances of the color solutions were taken in spectrophotometer against blank at 765 nm. The total phenolic contents were computed by the standard curve of gallic acid.

For flavonoids, 0.5 mL supernatant was mixed with 0.3 mL of 5% NaNO_2_ in a 10 mL tube and kept for 5 min. Then, 0.3 mL of 10% AlCl_3_ was added in tube. After 6 min, 2 mL of 1 M NaOH was added, and made the total volume up to 10 mL with distilled water and mixed well. The absorbances were observed in spectrophotometer (vs. blank) at 510 nm. The results were enumerated by quercetin standard curve.

### 3.5. Estimation of Carotenoids

For carotenoids, β-carotene and lycopene were estimated with little modification of previously described method [36]. Briefly, 1.0 g fresh tomato fruits were homogenized with 6 mL of acetone and hexane solution (2:3, *v*/*v*). Then, β-carotene and lycopene were estimated by measuring the absorbance in spectrophotometer at 453, 505, 645 and 663 nm, and calculated according to the following equations; β-carotene (mg/100 g FW) = [0.216 × OD_663_ − 1.220 × OD_645_ − 0.304 × OD_505_ + 0.452 × OD_453_] × 100; Lycopene (mg/100 g FW) = [−0.0458 × OD_663_ + 0.204 × OD_645_ − 0.304 × OD_505_ + 0.452 × OD_453_] × 100.

### 3.6. Quantification of Yield

The diameter and length of fruits were measured by using digital calipers (0–150 mm) to detect the changes of yield components. The fruits weight was estimated in digital weight scale (0.0001–200 g). Dry matter of fruits were determine by oven dry method at 65 °C.

### 3.7. Statistical Analysis

The statistical analysis was made by the IBM SPSS statistical program version 22 (SPSS-Inc., Chicago, IL, USA). The data were analyzed through one-way analysis of variance (ANOVA) and general linear method followed by Tukey’s test at *p* > 0.05 post hoc test for observation of the significance of differences between different treatments.

## 4. Conclusions

In our current study, we determined the effect of SAR stress and exogenous melatonin on antioxidant system in tomato fruits, which modulated the fruit qualities and health promoting antioxidant bioactive compounds in fruits and yield of tomatoes. We observed that SAR treatments in tomato plants increased the enzymatic antioxidant activities, qualities, health promoting bioactive compounds of fruits but greatly decreased the yield attributes of tomatoes. However, the application of melatonin in SAR-stressed plants exhibited more amplification on the enzymatic antioxidant system and different bioactive compounds in fruits, and also increased sharply the yield attributes of tomatoes. Our findings suggested that the application of melatonin improved the SAR-stressed tolerance in tomato fruits against oxidative damage which helped in improving the antioxidant system and health promoting antioxidant compounds in fruits as well as yield attributes. Consequently, the findings of the current study can be considered in developing new concerns for ensuring food security with considerable amount of health promoting antioxidant compounds in tomato fruits as well as other horticultural products in marginal areas where abiotic stress is a limiting factor to produce crops, fresh consumption and horticultural products processing industry in the future.

## Figures and Tables

**Table 1:** Effect of exogenous melatonin on carotenoids contents in fruits under SAR-stress condition.

Treatments	β-Carotene (mg/100 g FW)	Lycopene (mg/100 g FW)
Con	31.61 ± 1.19 ^c^	35.53 ± 1.04 ^c^
Con + Mel	34.61 ± 0.46 ^c^	36.67 ± 0.32 ^c^
SAR	41.96 ± 0.45 ^b^	42.83 ± 0.44 ^b^
SAR + Mel	45.87 ± 0.87 ^a^	47.32 ± 0.46 ^a^

Values indicate the averages of four replicates and ± indicates standard error. Different letters within the same parameter indicates significantly different at *p* ≤ 0.05 (Tukey’s test). Here, FW, Con, Mel, and SAR indicates fresh weight of fruit, control, melatonin, and simulated acid rain, respectively.

**Table 2:** Effect of exogenous melatonin on yield traits in tomatoes under SAR-stress condition.

Treatments	Diameter of Fruit (mm)	Length of Fruit (mm)	Number of Fruit	Average Weight g/Fruit	Dry Matter of Fruit (%)
Con	21.99 ± 0.34 ^a^	21.44 ± 0.18 ^a^	48.25 ± 1.75 ^a^	6.29 ± 0.15 ^a^	5.16 ± 0.06 ^c^
Con + Mel	22.73 ± 0.41 ^a^	22.13 ± 0.24 ^a^	51.25 ± 1.65 ^a^	6.50 ± 0.16 ^a^	5.24 ± 0.05 ^c^
SAR	16.79 ± 0.11 ^c^	16.35 ± 0.19 ^c^	22.75 ± 1.49 ^c^	3.95 ± 0.11 ^c^	5.87 ± 0.03 ^b^
SAR + Mel	19.35 ± 0.23 ^b^	19.12 ± 0.14 ^b^	38.25 ± 1.25 ^b^	5.03 ± 0.11 ^b^	6.11 ± 0.04 ^a^

Values indicate the averages of four replicates and ± indicates standard error. Different letters within the same parameter indicates significantly different at *p* ≤ 0.05 (Tukey’s test). Here, Con, Mel, and SAR indicates control, melatonin, and simulated acid rain, respectively.

## References

[1] Mirmiran P., Noori N., Zavareh M.B., Azizi F. (2009). Fruit and vegetable consumption and risk factors for cardiovascular disease. Metabolism.

[2] Garcia-Salas P., Morales-Soto A., Segura-Carretero A., Fernández-Gutiérrez A. (2010). Phenolic-compound-extraction systems for fruit and vegetable samples. Molecules.

[3] Buer C.S., Imin N., Djordjevic M.A. (2010). Flavonoids: New roles for old molecules. J. Integr. Plant Biol..

[4] De Pascual-Teresa S., Moreno D.A., García-Viguera C. (2010). Flavanols and anthocyanins in cardiovascular health: A review of current evidence. Int. J. Mol. Sci..

[5] Spencer J.P. (2010). The impact of fruit flavonoids on memory and cognition. Br. J. Nutr..

[6] Wu X., Gu L., Prior R.L., McKay S. (2004). Characterization of anthocyanins and proanthocyanidins in some cultivars of ribes, aronia, and sambucus and their antioxidant capacity. J. Agr. Food Chem..

[7] Torres C.A., Davies N.M., YANez J.A., Andrews P.K. (2005). Disposition of selected flavonoids in fruit tissues of various tomato (*Lycopersicon esculentum* Mill.) genotypes. J. Agr. Food Chem..

[8] Erba D., Casiraghi M.C., Ribas-Agustí A., Cáceres R., Marfà O., Castellari M. (2013). Nutritional value of tomatoes (*Solanum lycopersicum* L.) grown in greenhouse by different agronomic techniques. J. Food Compos. Anal..

[9] FAO (2014). Food and Agricultural Commodities Production for, Production of Tomato by Countries. http://faostat3.fao.org/home/E.

[10] Mu L., Fang L. (2016). Changes in tomato fruit quality and antioxidant enzyme activities under deficit irrigation and fertilizer application in a solar greenhouse in Northwest China. Commun. Soil Sci. Plant Anal..

[11] Duan L., Yu Q., Zhang Q., Wang Z., Pan Y., Larssen T., Tang J., Mulder J. (2016). Acid deposition in asia: Emissions, deposition, and ecosystem effects. Atmos. Environ..

[12] Bouwman A.F., Vuuren D.P.V., Derwent R.G., Posch M. (2002). A global analysis of acidification and eutrophication of terrestrial ecosystems. Water Air Soil Pollut..

[13] Kita I., Sato T., Kase Y., Mitropoulos P. (2004). Neutral rains at athens, greece: A natural safeguard against acidification of rains. Sci. Total Environ..

[14] Chen J., Li W., Gao F. (2010). Biogeochemical effects of forest vegetation on acid precipitation-related water chemistry: A case study in Southwest China. J. Environ. Monit..

[15] Wen K., Liang C., Wang L., Hu G., Zhou Q. (2011). Combined effects of lanthanumion and acid rain on growth, photosynthesis and chloroplast ultrastructure in soybean seedlings. Chemosphere.

[16] Zhang X., Du Y., Wang L., Zhou Q., Huang X., Sun Z. (2015). Combined effects of lanthanum (III) and acid rain on antioxidant enzyme system in soybean roots. PLoS ONE.

[17] Chen J., Wang W.-H., Liu T.-W., Wu F.-H., Zheng H.-L. (2013). Photosynthetic and antioxidant responses of *Liquidambar formosana* and *Schima superba* seedlings to sulfuric-rich and nitric-rich simulated acid rain. Plant Physiol. Biochem..

[18] Kováčik J., Klejdus B., Bačkor M., Stork F., Hedbavny J. (2011). Physiological responses of root-less epiphytic plants to acid rain. Ecotoxicology.

[19] Dolatabadian A., Sanavy S.A.M.M., Gholamhoseini M., Joghan A.K., Majdi M., Kashkooli A.B. (2013). The role of calcium in improving photosynthesis and related physiological and biochemical attributes of spring wheat subjected to simulated acid rain. Physiol. Mol. Biol. Plants.

[20] Debnath B., Irshad M., Mitra S., Li M., Liu S., Rizwan H.M., Pan T., Qiu D. (2018). Acid rain deposition modulates photosynthesis, enzymatic and non-enzymatic antioxidant activities in tomato. Int. J. Environ. Res..

[21] Dursun A., Yildirim E., Güvenc I., Kumlay A.M. (2002). Effects of Simulated Acid Rain on Plant Growth and Yield of Tomato (Lycopersicon Esculentum).

[22] Zhai Y., Yang Q., Hou M. (2015). The effects of saline water drip irrigation on tomato yield, quality, and blossom-end rot incidence—A 3a case study in the south of China. PLoS ONE.

[23] Zushi K., Ono M., Matsuzoe N. (2014). Light intensity modulates antioxidant systems in salt-stressed tomato (*Solanum lycopersicum* L. Cv. Micro-tom) fruits. Sci. Hortic..

[24] Murshed R., Lopez-Lauri F., Sallanon H. (2013). Effect of water stress on antioxidant systems and oxidative parameters in fruits of tomato (*Solanum lycopersicon* L, cv. Micro-tom). Physiol. Mol. Biol. Plants.

[25] Moyle L.C. (2008). Ecological and evolutionary genomics in the wild tomatoes (*Solanum* sect. *Lycopersicon*). Evolution.

[26] Tan D.-X., Hardeland R., Manchester L.C., Korkmaz A., Ma S., Rosales-Corral S., Reiter R.J. (2011). Functional roles of melatonin in plants, and perspectives in nutritional and agricultural science. J. Exp. Bot..

[27] Arnao M.B., Hernández-Ruiz J. (2015). Functions of melatonin in plants: A review. J. Pineal Res..

[28] Galano A., Tan D.X., Reiter R.J. (2011). Melatonin as a natural ally against oxidative stress: A physicochemical examination. J. Pineal Res..

[29] Debnath B., Hussain M., Irshad M., Mitra S., Li M., Liu S., Qiu D. (2018). Exogenous melatonin mitigates acid rain stress to tomato plants through modulation of leaf ultrastructure, photosynthesis and antioxidant potential. Molecules.

[30] Okazaki M., Ezura H. (2009). Profiling of melatonin in the model tomato (*Solanum lycopersicum* L.) cultivar micro-tom. J. Pineal Res..

[31] Sun Q., Zhang N., Wang J., Zhang H., Li D., Shi J., Li R., Weeda S., Zhao B., Ren S. (2014). Melatonin promotes ripening and improves quality of tomato fruit during postharvest life. J. Exp. Bot..

[32] Feng X., Wang M., Zhao Y., Han P., Dai Y. (2014). Melatonin from different fruit sources, functional roles, and analytical methods. Trends Food Sci. Technol..

[33] Keutgen A.J., Pawelzik E. (2007). Modifications of strawberry fruit antioxidant pools and fruit quality under nacl stress. J. Agr. Food Chem..

[34] Cui G., Zhao X., Liu S., Sun F., Zhang C., Xi Y. (2017). Beneficial effects of melatonin in overcoming drought stress in wheat seedlings. Plant Physiol. Biochem..

[35] Luthria D.L., Mukhopadhyay S., Krizek D.T. (2006). Content of total phenolics and phenolic acids in tomato (*Lycopersicon esculentum* Mill.) fruits as influenced by cultivar and solar uv radiation. J. Food Compos. Anal..

[36] Vinha A.F., Alves R.C., Barreira S.V., Castro A., Costa A.S., Oliveira M.B.P. (2014). Effect of peel and seed removal on the nutritional value and antioxidant activity of tomato (*Lycopersicon esculentum* L.) fruits. LWT Food Sci. Technol..

[37] Saleem A., Ashraf M., Akram N., Al-Qurainy F. (2012). Salinity-induced changes in key anti-oxidant enzyme activities and in the levels of some anti-oxidants, osmo-protectants, inorganic ions, and chlorophyll pigments in okra fruit (*Abelmoschus esculentus* L.). J. Hortic. Sci. Biotech..

[38] Turk H., Erdal S., Genisel M., Atici O., Demir Y., Yanmis D. (2014). The regulatory effect of melatonin on physiological, biochemical and molecular parameters in cold-stressed wheat seedlings. Plant Growth Regul..

[39] Borghesi E., González-Miret M.L., Escudero-Gilete M.L., Malorgio F., Heredia F.J., Meléndez-Martínez A.J. (2011). Effects of salinity stress on carotenoids, anthocyanins, and color of diverse tomato genotypes. J. Agr. Food Chem..

[40] Bradford M.M. (1976). A rapid method for the quantitation of microgram quantities of protein utilizing the principle of protein-dye binding. Anal. Biochem..

[41] Yemm E., Willis A. (1954). The estimation of carbohydrates in plant extracts by anthrone. Biochem. J..

[42] Ahmed I.M., Cao F., Zhang M., Chen X., Zhang G., Wu F. (2013). Difference in yield and physiological features in response to drought and salinity combined stress during anthesis in tibetan wild and cultivated barleys. PLoS ONE.

[43] Benzie I.F., Strain J.J. (1996). The ferric reducing ability of plasma (frap) as a measure of “antioxidant power”: The frap assay. Anal. Biochem..

